# Supplementary material for: Temozolomide Treatment Increases Fatty Acid Uptake in Glioblastoma Stem Cells
Source: Cancers (Basel). 2020 Oct 26;12(11):3126. doi: 10.3390/cancers12113126 (PMC7693784; doi:10.3390/cancers12113126)
Supplement: Supplementary file 1 [file cancers-12-03126-s001.pdf]

Supplementary Material: Temozolomide Treatment Increases Fatty Acid Uptake in Glioblastoma Stem Cells

Seamus Caragher, Jason Miska, Jack Shireman, Cheol H. Park, Megan Muroski, Maciej S. Lesniak and Atique U. Ahmed

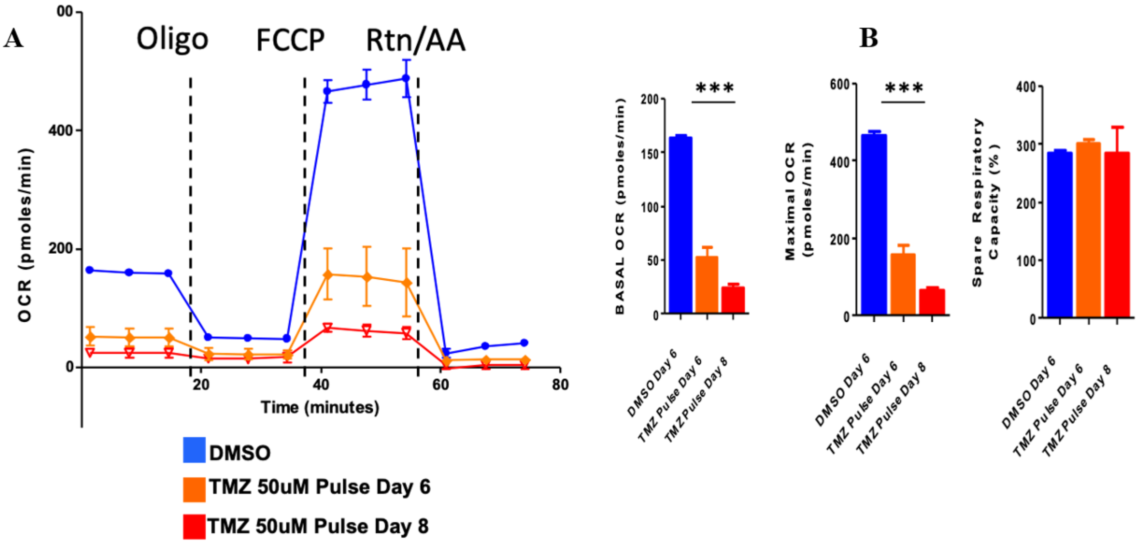

Figure S1. Pulsatile TMZ treatment induces GBM cells into mitochondrial metabolic senescence. (**A**) Representative tracing of metabolic analysis. Patient derived xenograft (PDX) GBM 43 cells were cultured with temozolomide (TMZ, 50 μM) or equimolar DMSO for 18 h, after which all cells were washed and fresh media added. Cells were then left unperturbed for 6 or 8 days and metabolic phenotype assessed by Seahorse. (**B**) Analysis reveal that cells treated with TMZ became metabolically quiescent after pulsatile treatment. Error bars show standard deviation. Comparison was performed using Student's *t*-Test. *** $p < 0.001$.

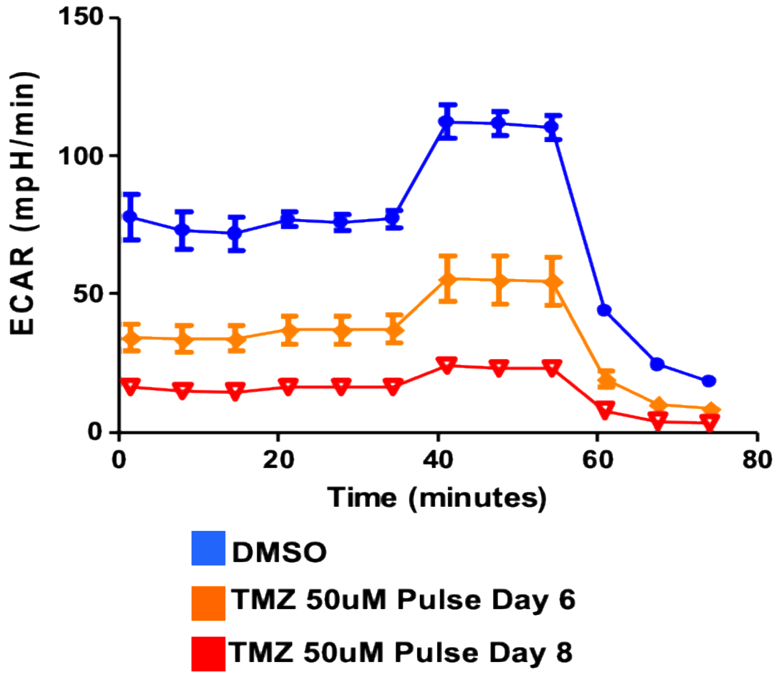

Figure S2. Withdrawal of therapeutic stress induces GBM cells into glycolytic senescence. Representative tracing of metabolic analysis. Patient derived xenograft (PDX) GBM 43 cells were cultured with temozolomide (TMZ, 50 µM) or equimolar DMSO for 18 h, after which all cells were washed and fresh media added. Cells were then left unperturbed for 6 or 8 days and metabolic phenotype assessed by Seahorse. Analysis reveal that cells treated with TMZ significantly reduce glycolytic activity following pulsatile chemotherapy exposure.

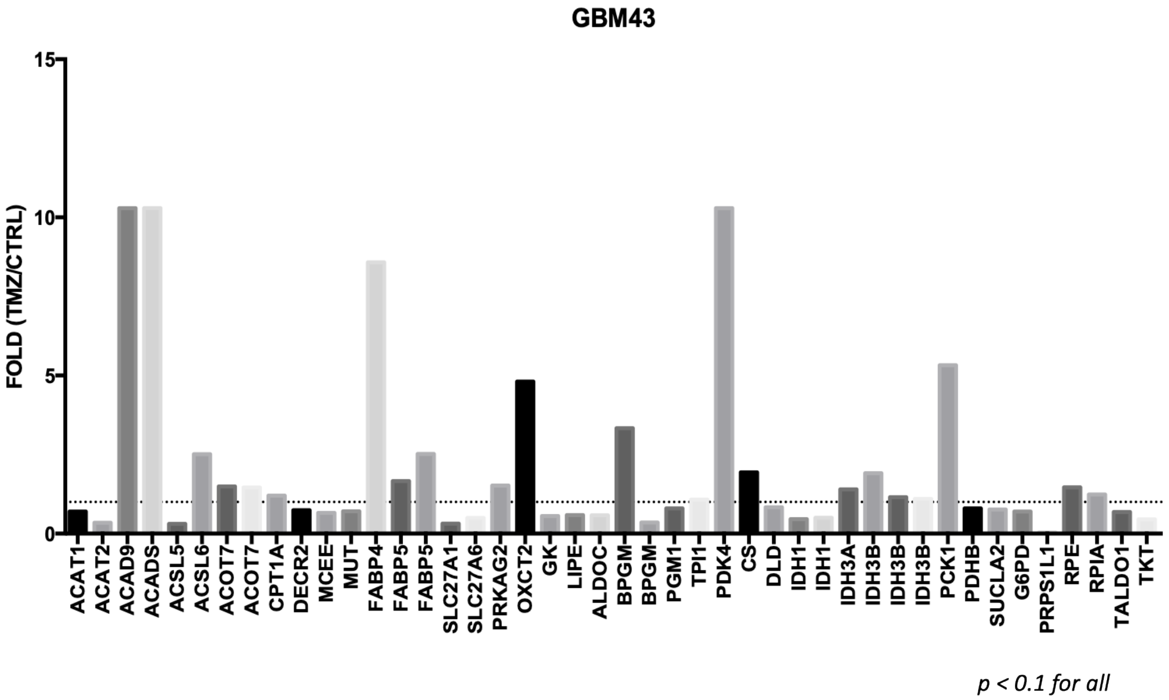

Figure S3. Microarray analysis revealed upregulation of a number of gene transcripts related to metabolism following chemotherapeutic stress. GBM43 were treated with 50 µM TMZ. After 8 days, cells were collected, and mRNA extracted. Microarray was performed with Affymetrix 1300 platform. These genes were all those found to be significant with a p value less than 0.1. Dashed line equals fold change of 1.

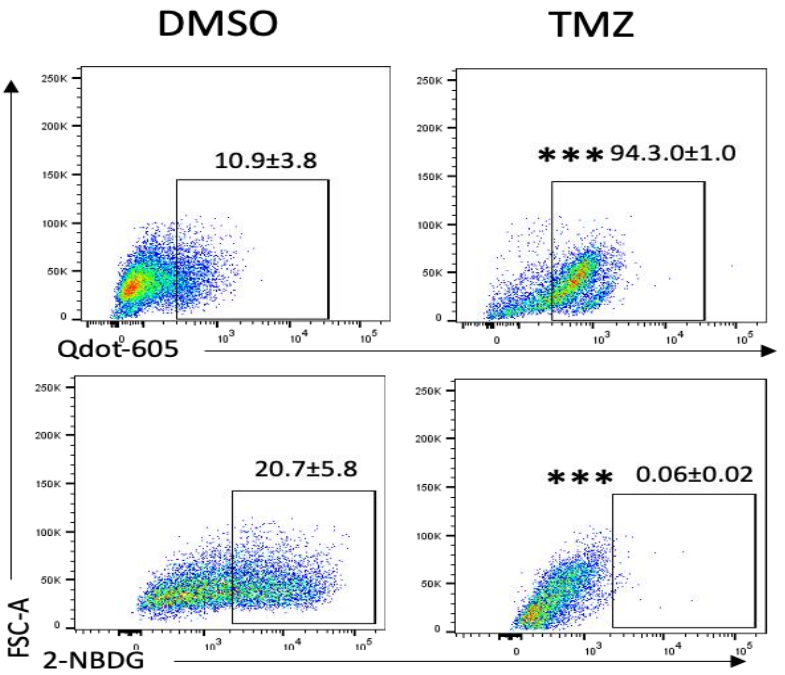

Figure S4. Temozolomide-induced stress alters GBM metabolism. Representative image of fatty acid uptake (Qdot-605) and glucose uptake (2-NBDG) in GBM43 cells treated with DMSO or 50 µM TMZ. CD133 specific populations are shown in Figure 4. Error bars show standard deviation across multiple replicates. Comparison was performed using Student's *t*-Test. *** $p < 0.001$.

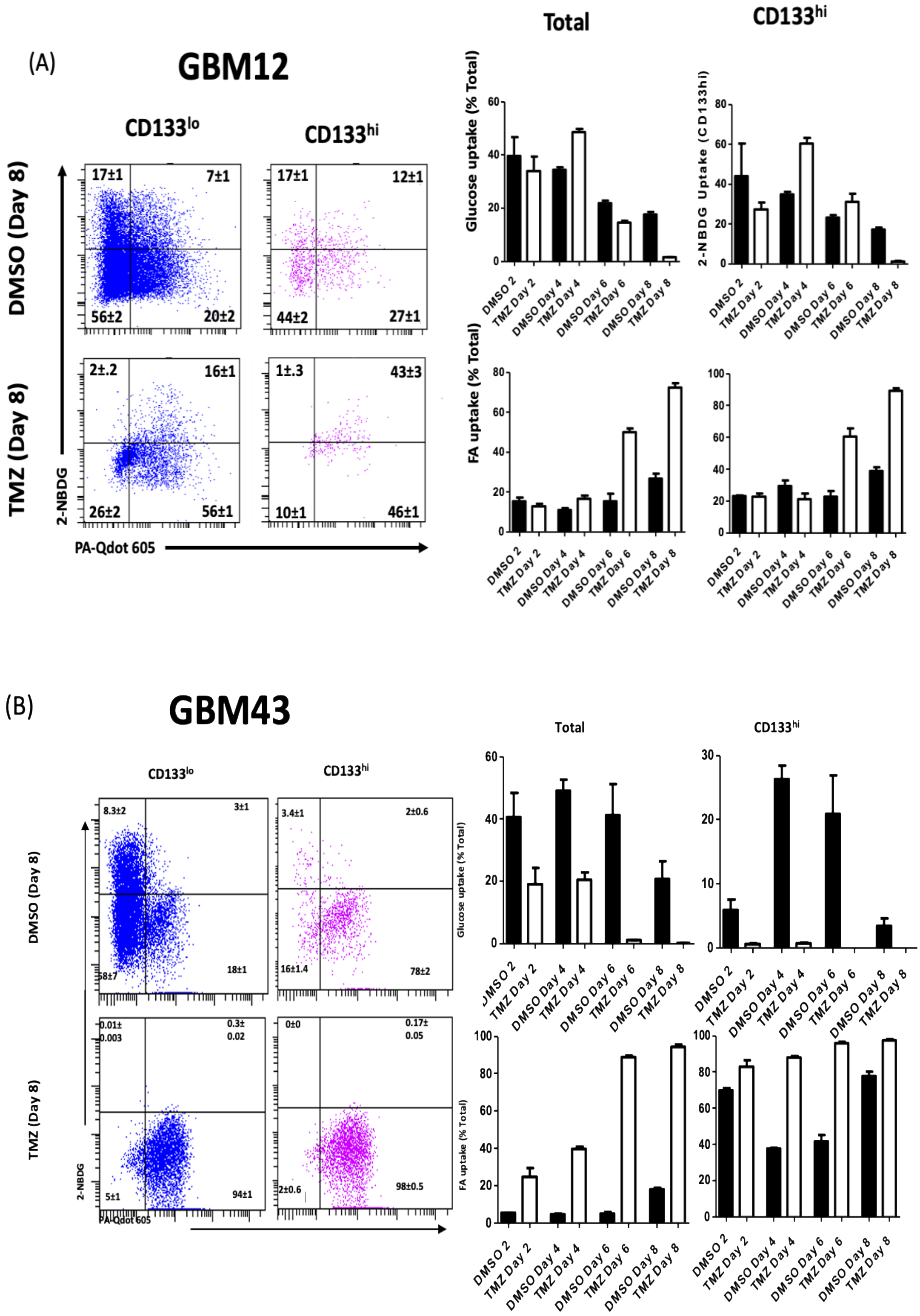

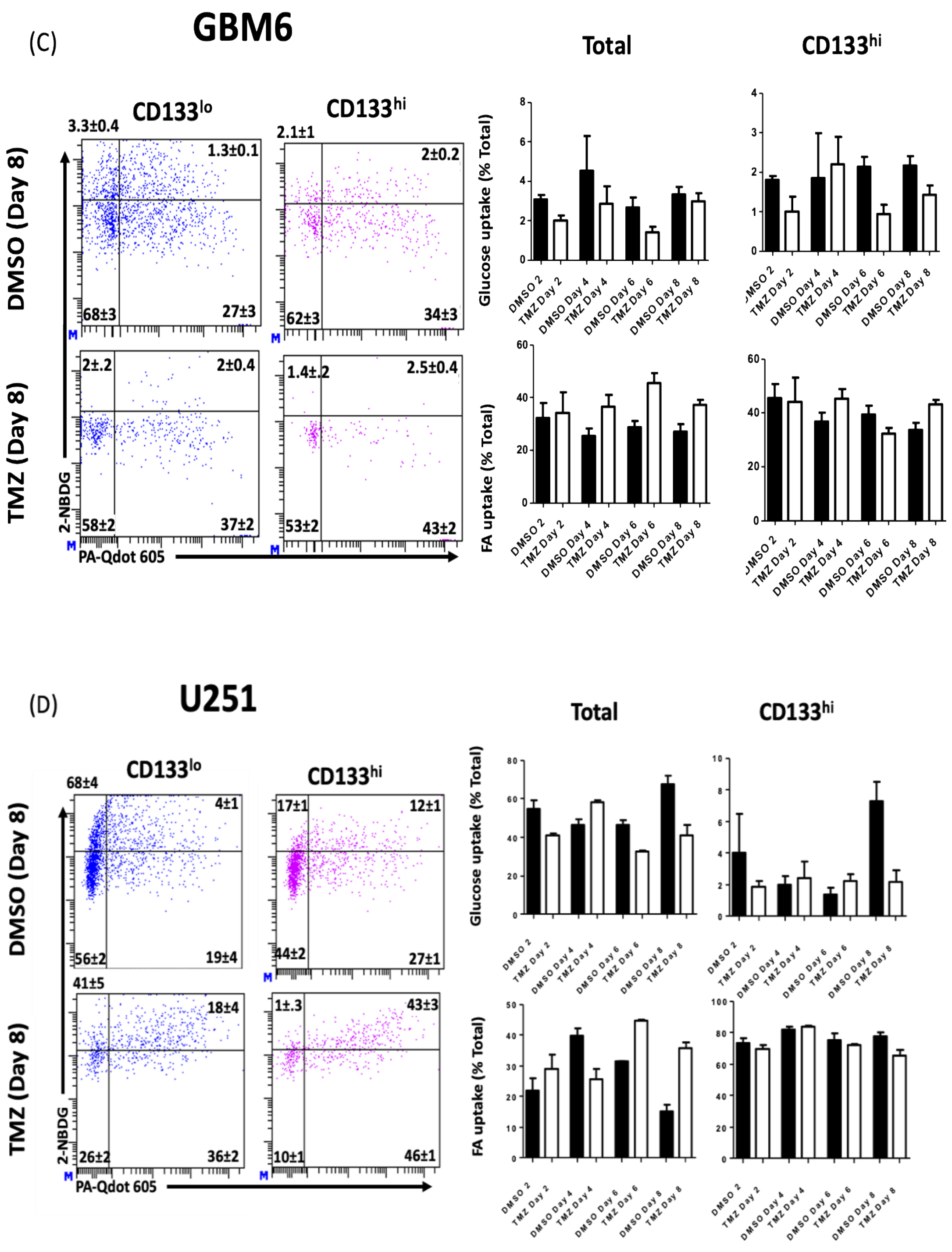

Figure S5. Chemotherapeutic stress alters GBM metabolism in a range of tumor subtypes. (**A–D**) Multiple GBM cell lines were treated with TMZ (50 μM) or equimolar DMSO for 2, 4, 6, or 8 days, after which uptake of glucose and fatty acid were analyzed via FACS analysis, as in Figure 4/5. Each panel provides representative FACS tracings and cell line specific analysis. Error bars show standard deviation.

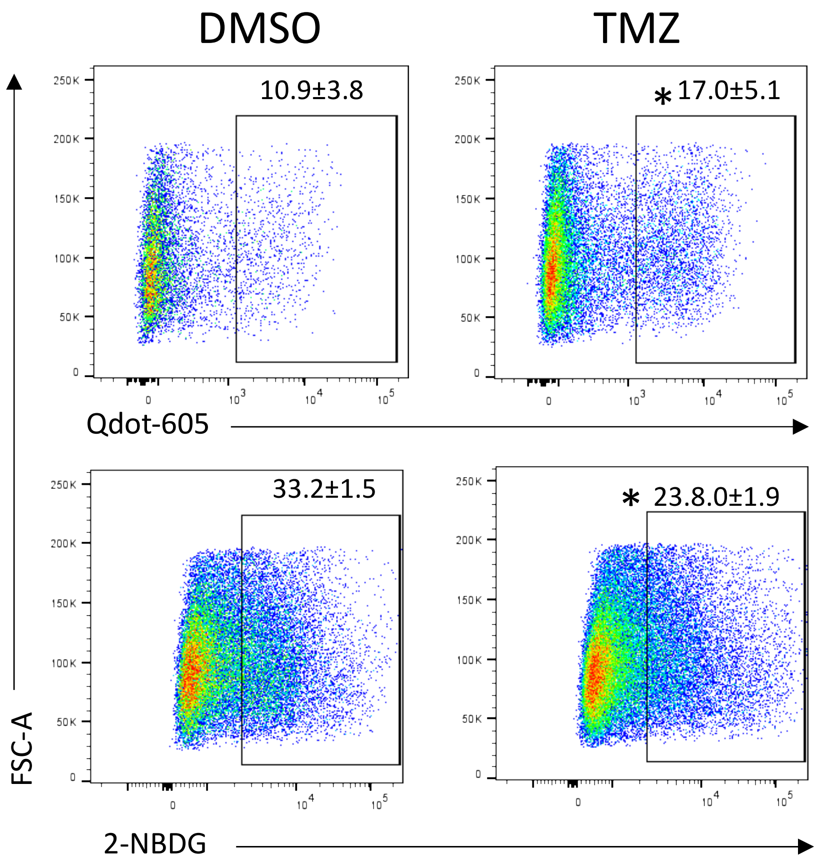

Figure S6. Temozolomide-induced stress alters GBM metabolism in vivo. Representative image of fatty acid uptake (Qdot-605) and glucose uptake (2-NBDG) in GBM43 cells from murine intracranial xenografts treated with DMSO or 50 µM TMZ. CD133 specific populations are shown in Figure 6. Error bars show standard deviation across multiple replicates. Comparison was performed using Student's *t*-Test. * $p < 0.05$.
